# Lethal coalitionary attacks of chimpanzees (*Pan troglodytes troglodytes*) on gorillas (*Gorilla gorilla gorilla*) in the wild

**DOI:** 10.1038/s41598-021-93829-x

**Published:** 2021-07-19

**Authors:** Lara M. Southern, Tobias Deschner, Simone Pika

**Affiliations:** 1 Institute of Cognitive Science, Comparative BioCognition, University of Osnabrück, Artilleriestrasse 34, 49076 Osnabrück, Germany; 2 Max Planck Institute for Evolutionary Anthropology, Interim Group Primatology, Deutscher Platz 6, 04103 Leipzig, Germany

**Keywords:** Social evolution, Animal behaviour, Behavioural ecology

## Abstract

Intraspecies violence, including lethal interactions, is a relatively common phenomenon in mammals. Contrarily, interspecies violence has mainly been investigated in the context of predation and received most research attention in carnivores. Here, we provide the first information of two lethal coalitionary attacks of chimpanzees (*Pan troglodytes troglodytes*) on another hominid species, western lowland gorillas (*Gorilla gorilla gorilla*), that occur sympatrically in the Loango National Park in Gabon. In both events, the chimpanzees significantly outnumbered the gorillas and victims were infant gorillas. We discuss these observations in light of the two most widely accepted theoretical explanations for interspecific lethal violence, predation and competition, and combinations of the two-intraguild predation and interspecific killing. Given these events meet conditions proposed to trigger coalitional killing of neighbours in chimpanzees, we also discuss them in light of chimpanzees’ intraspecific interactions and territorial nature. Our findings may spur further research into the complexity of interspecies interactions. In addition, they may aid in combining field data from extant models with the Pliocene hominid fossil record to better understand behavioural adaptations and interspecific killing in the hominin lineage.

Intraspecies violence resulting in lethal injuries occurs in a variety of mammal species^1^, and has been suggested to follow patterns explicable by kin selection^2,3^ and evolutionary game theory^4^.

Concerning our closest living relatives, the great apes, intraspecific killing has frequently been reported across multiple chimpanzee (*Pan troglodytes*) communities e.g.,^5–7^ and gorilla (*Gorilla gorilla*)^8–10^ groups. However, it is nearly absent in bonobos (*Pan paniscus*) (but see^11^) and orangutans (*Pongo* ssp.)^12^. Rates of intraspecific killings vary considerably among chimpanzee communities, with adult males being both the main attackers and the main victims^11^. The majority of killings involve intercommunity rather than intracommunity attacks, and most often are made by coalitions of males during territorial boundary patrols^5,13^. During these patrols, chimpanzees travel to the periphery of the territory to search for signs of members of other communities or may even make deep incursions into neighbouring communities involving lethal coalitionary attacks^13–15^. The latter has been associated with fission–fusion social systems and has spurred considerable research attention, suggesting functional parallels and evolutionary continuities between chimpanzee violence and lethal intergroup raiding in humans^6,16,17^.

In contrast, intraspecific killings in gorillas have almost exclusively been observed in intergroup encounters^10,18^ (but see^9^). Gorillas (genus *Gorilla*) are as genetically distant from chimpanzees (genus *Pan*) as they are from humans (genus *Homo*), and are thought to have separated from a shared ancestor around eight million years ago^19^. Across their geographic range, gorillas live in cohesive social groups consisting of one or more adult males, adult females, and their offspring^8,20–22^. Unlike chimpanzees, the home ranges of neighbouring gorilla groups overlap greatly, but intergroup encounters also range from non-agonistic affiliative encounters to coalitionary agonistic interactions involving physical violence, infanticide^8–10^ and occasionally even fatal injuries^18,23–25^ to adult males. The majority of studies have, however, only focused on one eastern sub-species (*Gorilla beringei beringei*), limiting an in-depth understanding of the behavioural diversity of gorillas. For instance, a recent study on western lowland gorillas (*Gorilla gorilla gorilla*) suggested that groups may show high levels of territoriality and actively defend core regions of their home ranges against neighbours^26^.

Interspecific violence including lethal encounters has been reported across a variety of species and taxa and has been traditionally categorized as predation or competitive killing^27,28^. Predation is commonly viewed as an organism killing another organism for nutritional purposes^29^. Interspecific competition can involve (i) exploitative competition (in which a species indirectly competes with other species for common resources), and (ii) interference competition (in which a species attempts to free resources by interfering directly with another species in the form of aggression, intimidation, harassment, competitive exclusion, or killing of the interspecific competitor)^27,30^. Killing amongst interspecific competitors—viewed as a combination of competition and predation by some^31^—has recently gained a lot of research attention with scholars distinguishing between intraguild predation (IGP) and interspecific killing (IK)^31–33^. IGP is the killing and eating of species of the same “guild” that use similar, often limiting, resources (and are thus potential competitors) and has been shown to play a crucial role in carnivores^31,32^. A guild includes all species exploiting similar resources, regardless of their nutrition mode, ecology, or taxonomic position^31^. IGP results in an immediate nutritional gain for one participant, the predator, whilst, in contrast, IK refers to the killing of potentially competing species without any immediate nutritional gain to the aggressor^33^.

Concerning great apes, interspecific violence in the form of predation and hunting has been observed in bonobos^34,35^ and chimpanzees e.g.,^36,37^. In bonobos, both sexes engage in pursuing and hunting^34,35,37^, and hunts are individualistic and opportunistic (i.e. they do not involve previous searching or tracking behaviour^35^). Contrarily, hunting in chimpanzees is predominantly a male activity^38–40^, and cooperative hunting has been observed at some sites^41^. Although hunting is often opportunistic^37^, chimpanzees at Taï, Taï National Park, Cote D’Ivoire^41^ and Ngogo, Kibale National Park, Uganda^38,42^ have been observed to search actively for prey and listen for distinct vocalizations. Chimpanzees prey upon a broad variety of taxa including birds, insects, non-primate mammals, monkeys, and reptiles e.g.,^13,40–43^, with prey preferences differing between chimpanzee populations based on local availability^37,44,45^.

Here, we report the first observations of two lethal coalitionary attacks of chimpanzees (*Pan troglodytes troglodytes*) on gorillas (*Gorilla gorilla gorilla*) living in the Loango National Park, Gabon. Chimpanzees range across Eastern and Central Africa, and live sympatrically with gorillas (*Gorilla gorilla* spp.) in some areas^46^. Population estimates vary widely depending on how they are calculated^47,48^, making cross site comparisons so far difficult and largely inaccurate. Additionally, surveys of great apes have been challenging due to their low densities, cryptic nature and difficulties in accessing the habitats they live in e.g.,^49^. The densities of gorillas and chimpanzees in a portion of Loango National Park that includes our study area (101–123 km^2^) were estimated using genetic capture–recapture methods, with 0.8–1.1 chimpanzees and 1.2–1.4 gorillas per km^2^ ^50,51^. These estimations of chimpanzee density are moderate compared to other field sites where the two species occur sympatrically, while the gorilla densities are significantly lower (but see variety of methods used, e.g.,^48,52–54^).

We discuss the observed lethal events in relation to the two most widely accepted theoretical explanations for interspecific violence, predation and competition, as well as combinations of the two—intraguild predation (IGP) and interspecific killing (IK). Furthermore, we pinpoint similarities to intraspecific killing and the territorial behaviour of chimpanzees.

## Results

### Overview

**Figure 1:**
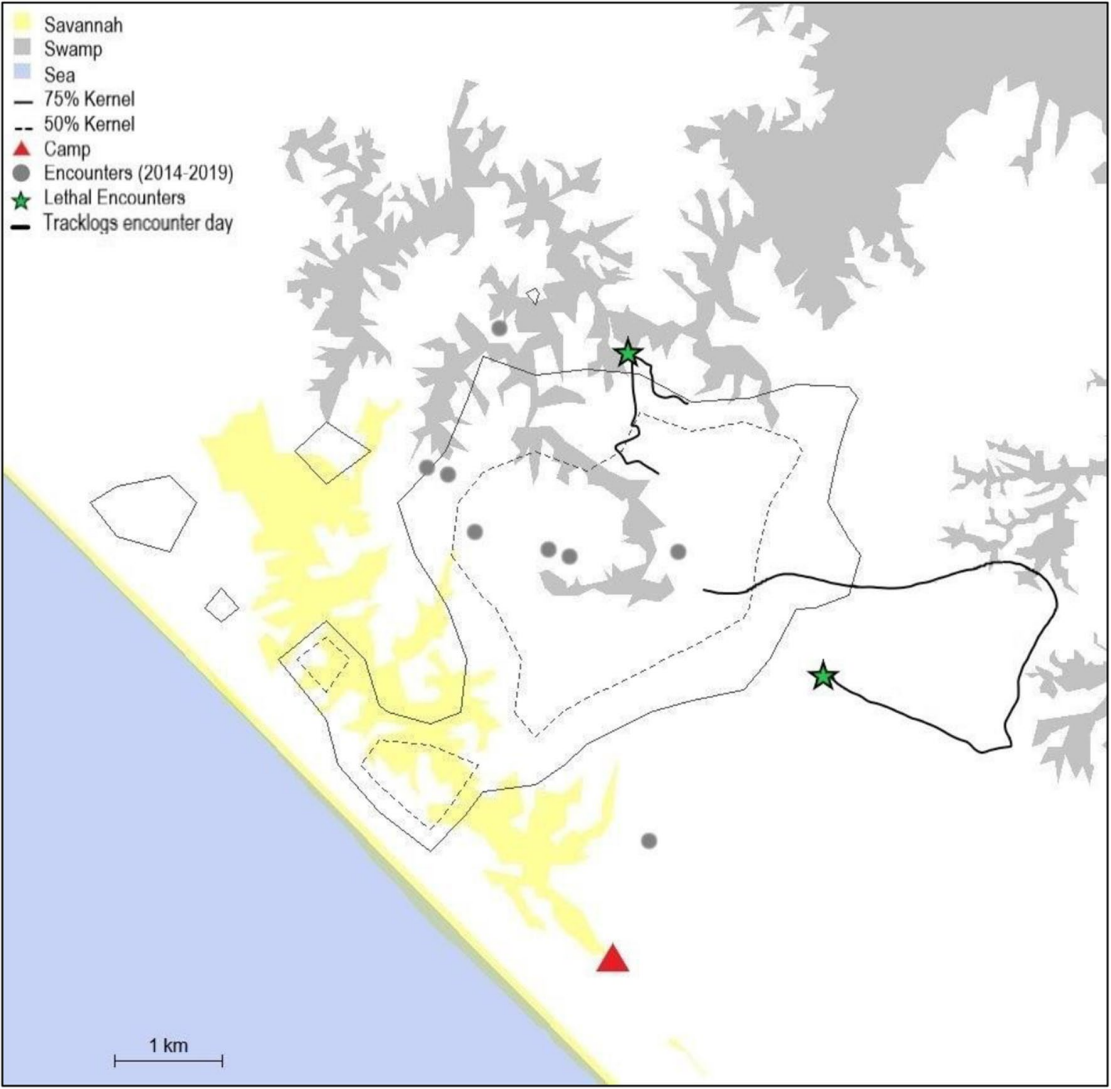
Map of the study area and location of events. The locations of the two lethal encounters of the 06/02/2019 and the 11/12/2019 are marked with green stars. Nine previous encounters with gorillas (2014–2019) are marked with eight grey circles since two event locations were identical) characterized by peaceful behaviour and, in two cases, co-feeding. The 50% and 75% density isopleth^55^ of the home range and travel paths based on tracklog data from the two encounter days are marked in broken grey, solid grey and black lines. The figure was generated in R (version 3.6.3, R Core team, https://www.R-project.org/)^56^ using the package adehabitatHR^57^.

Between 2014 and 2018, we observed nine direct interactions between individuals of the Rekambo community and unhabituated gorillas (N = 9; see Fig. 1). These events were always peaceful, and occasionally involved co-feeding in fruiting trees (N = 2). In 2019 however, we observed two encounters resulting, in both cases, in coalitionary lethal attacks. The first encounter involved a party of 27 chimpanzees and a group of five gorillas. The second involved a party of 27 chimpanzees and a group of seven gorillas (see Table 1, and video clips 1 and 2 in the SA). The first event occurred after a territorial patrol during which the males made a deep incursion into a neighbouring chimpanzee territory. The second event happened at the start of a suspected territorial boundary patrol. Both events took place on the outer boundaries of the Rekambo territory (see Fig. 1). The main aggressors in both events were adult male chimpanzees (for details of involvement see Table 1).

**Table 1:** Chimpanzees observed during the gorilla encounters.

Individual	Sex	Age class	Role during encounter 1	Role during encounter 2
Arnold	M	Adult	Not active	Active (APC)
Chenge	M	Adult	Active (APC)	Active (APC)
Chinois	M	Adult	Active (NPC)	Active (NPC)
Freddy	M	Adult	Active (APC)	Active (APC)
Gump	M	Adult	Active (APC)	Active (APC)
Littlegrey	M	Adult	Active (APC)	Active (APC)
Louis	M	Adult	Active (APC)	Active (NPC)
Ngonde	M	Adult	Active (APC)	Active (APC)
Orian	M	Adult	Not active	Active (APC)
Pandi	M	Adult	Active (APC)	Active (APC)
Thea	M	Adult	Active (APC)	Active (APC)
Carol	F	Adult	Not active	Not active
Emmie	F	Adult	N/A	Not involved
Ida	F	Adult	Not involved	N/A
Joy	F	Adult	Not involved	Not involved
Mimi	F	Adult	Not involved	Active (APC)
Onome	F	Adult	Not involved	Not involved
Roxy	F	Adult	Active (APC)	Active (APC)
Spock	F	Adult	N/A	Not involved
Suzee	F	Adult	Not involved	N/A
Cesar	M	Adolescent	Active (PC)	Active (PC)
Sia	M	Adolescent	Active (PC)	N/A
Clessia	F	Adolescent	Active (PC)	N/A
Gia	F	Adolescent	Active (APC)	Active (PC)
Greta	F	Adolescent	N/A	Active (PC)
Harouenne	F	Adolescent	Active (PC)	N/A
Queliba	F	Adolescent	N/A	Active (PC)
Ernest	M	Juvenile	N/A	Not involved
Moana	M	Juvenile	Not involved	Active (PC)
Ismael	M	Infant	Not involved	N/A
Jambo	M	Infant	N/A	Not involved
Caya	F	Infant	N/A	Not active
Madiba	F	Infant	Not involved	Not active
Sassandra	F	Infant	Not involved	N/A

Members of the Rekambo chimpanzee community present during encounter 1 (N = 27) and encounter 2 (N = 27) as a function of name, sex (M = male; F = female), age class and role. The role describes the observed activity of individuals during the encounters: *N/A* not present, *not involved* seen only at the start and after the encounter, therefore their presence is inferred, *not active* observed during the encounter but no active role in the attack, *active-no physical contact* (NPC) active role in the attack (e.g. charging, chasing) but no direct contact with a gorilla, *active-aggressive physical contact* (APC) active role in the attack with direct aggressive behaviours towards a gorilla individual (e.g., hitting, biting, dragging), *active-physical contact* (PC) present throughout attack but direct contact only through handling of already deceased infant gorilla (e.g., touching, sniffing, handling).

The two encounters lasted 52 and 79 min, respectively, involved both contact and non-contact aggressions and coalitionary displays from chimpanzees towards gorillas***.*** The gorillas counter attacked and defended themselves using contact aggressions, displays and threat gestures. During the first encounter the silverback was present for 14 min, whilst in the second encounter the silverback was present for 10 min. The first encounter resulted in one dead gorilla infant and three injured chimpanzees; the second resulted in one dead gorilla infant. While there was no indication of consumption of the dead gorilla infant in the first encounter, the infant in the second encounter was almost entirely consumed by one adult chimpanzee female.

#### Lethal encounter 1

On February 6th 2019, two research teams followed a party of chimpanzees (N = 27; see Table 1) of the Rekambo community. The party displayed behaviours observed during territorial patrols and exited their territory at approximately 11:50 (see tracklog in Fig. 1). At 16:45, after not encountering any direct or indirect signs of a neighbouring chimpanzee community, the entire party returned towards the eastern border of their territory and split into sub-groups of 18 and 9 individuals. The vegetation in this area is dense and visibility was limited.

At 17:01, the larger chimpanzee party (N = 18) encountered a group of gorillas (estimated N = 5; 1 silverback, 3 adult females, 1 infant) in a thicket of approximately 64 square meters. A first chimpanzee scream was followed by a succession of chimpanzee screams and barks, and gorilla barks and roars (for definition of call types see^13,58^). At 17:13, the silverback charged an adolescent female chimpanzee, Gia, knocking her into the air. At 17:15, a group of approximately nine male chimpanzees (adults and adolescents), and at least one adult female chimpanzee surrounded the silverback, and repeatedly jumped down on and hit him whilst screaming and barking. The silverback retreated to a distance of approximately 30 m with all other members of his group.

At 17:22, one adult male chimpanzee, Littlegrey, was observed sitting on the ground holding a gorilla infant in front of him. The infant emitted distress vocalizations but did not move. Between 17:22 and 17:26 the infant was inspected by three adult male chimpanzees, Gump, Ngonde and Thea, and two adolescents, Cesar and Sia. The infant was then taken by Gump but quickly retrieved by Littlegrey. At 17:26, Littlegrey sniffed the infant, placed it on the ground in front of him and hit the infant three times with his right hand. At this point the gorilla infant was still alive since short squeaks and whimpering sounds could be heard. At 17:27, and directly following a distress vocalization of the infant gorilla, a whimper of a different gorilla was heard close by (approximately 20 m).

At 17:28, Ngonde grabbed the infant gorilla and pulled it by its right foot for approximately three meters whilst the infant omitted distress vocalizations. At 17:30, Clessia, an adolescent female, took the gorilla infant from Ngonde, sniffed the body, and held it with both hands while lying on her back. At 17:36, the gorilla infant stopped vocalizing, and no further vital signs were observed. Between 17:36 and 18:05, Clessia continued to hold and periodically play with the now lifeless body of the gorilla. At 17:53, a chest beat was heard from a gorilla (at approximately 40 m), but subsequently no further sound, vocalization or indication of gorilla presence was noted. Clessia was still in possession of the gorilla body when the observers left at 18:15. The adolescent chimpanzee female Gia, was severely injured, with suspected internal bruising, and two adult males showed minor abrasions (for further details see Supplementary Information).

#### Lethal encounter 2

On December 11th 2019, two observation teams had been following 27 chimpanzees moving northwest towards the northern border of their territory. Given the direction and the surreptitious behaviour of the individuals present, including frequent sniffing of the ground and vegetation^15^, a territorial patrol seemed imminent.

At 12:26, Freddy suddenly stopped, became pilo-erect, and produced alarm barks. The chimpanzees around him then stopped travelling and started alarm barking as well. There was movement observed in a large tree at approximately 40 m. At 12:28, observers identified an adult female gorilla in the canopy. The chimpanzees then moved towards the tree (not a known fruiting tree species for either ape species) looking up into the canopy.

At 12:28, gorilla barks and chest beats were heard and the team observed six additional gorillas in the tree: a silverback, two adult females with dependent infants, and one juvenile gorilla. At 12:30, the majority of the chimpanzees started to climb up into the surrounding trees, while approximately four adult male chimpanzees remained on the ground. At 12:33, the silverback discovered the human observers on the ground in a distance of approximately 30 m to the base of the tree and started barking. In response, the two observer teams increased their distance to the tree from 30 to 60 m. One adult male chimpanzee, Chenge, climbed further up the tree with the gorillas, and stopped within five meters of the silverback and one adult female gorilla with an infant (AF1 and I1). All visible gorillas started to emit alarm barks, and the silverback and the two adult females with their infants moved higher up into the canopy. At 12:36, the silverback rapidly climbed down the tree and fled. The chimpanzees continued barking but did not follow him.

At 12:37, one of the two adult gorilla females (AF2) with her infant (I2) on her belly climbed down the tree with a group of chimpanzees surrounding her while barking, screaming, charging and branch shaking. Two adult chimpanzee males, Pandi and Thea dragged branches and displayed at the female. She held her infant on her belly and barked, the infant was also barking and screaming. Thea positioned himself several times directly in front of the female, and tried to grab the infant but he did not succeed. Gump managed to temporarily pull the infant from the belly of the female but she managed to pull her infant back. At 12:40, the female gorilla and her infant managed to escape the group of chimpanzees. The chimpanzees did not follow her but instead directed their attention to an area approximately 20 m away across a swamp where gorilla screams and barks could be heard.

A third separate observation team, who arrived later had a better view of the following events. Between 12:41 and 12:48, they observed one adult female gorilla (AF1) with a smaller infant (I1) in a tree with eight adult chimpanzee males (Chenge, Gump, Littlegrey, Louis, Ngonde, Orian, Pandi, and Thea), and one adolescent female (Greta) sitting in trees around her. No other gorilla was visible at this point; however, chest beats and barks were heard from the surrounding canopy. Littlegrey, Louis, and Pandi displayed repeatedly shaking branches, and all chimpanzees continuously emitted alarm barks. The gorilla female screamed and barked as soon as any chimpanzee undercut a distance of approximately five meters. At 12:48, four chimpanzee males (Ngonde, Orian, Pandi and Thea) started to chase the female first further up the tree, and then down the tree while barking and screaming (see video clips in SA). During approaches, the gorilla female (AF1) waved her arms and screamed towards the chimpanzees, while holding her infant (I1) to her belly, and simultaneously trying to move out of proximity of the chimpanzees. At 12:49, the gorilla female moved rapidly down the tree with her infant (I1) on her belly into a tangle of lianas. At 12:50, the gorilla female (AF1) was seen, without her infant (I1), climbing up a nearby tree, observed by several chimpanzees (Freddy, Gump, Louis, Mimi, Moana, and Thea). She managed to flee via the canopy.

At 12:51, an adolescent chimpanzee, Cesar, was seen holding the body of a dead infant gorilla (I1), which had a large open cut in the stomach with the intestines partially hanging out. Gump then approached Cesar, grabbed the lifeless gorilla, and ran away with it. At 12:53, chest beats of a gorilla were heard at a distance of approximately 75 m. At this time, all other chimpanzees of the party were dispersed around the encounter site, and rested either on the ground or up in the trees. The observers were able to locate the lifeless gorilla infant again at 12:57, when Gump, followed by Greta, climbed a tree holding it. At 13:00, Greta was observed holding the body, and eating small pieces of meat from the extremities in proximity to one adult female, Onome and an adolescent female, Queliba. There were no begging behaviours observed from the females in proximity and Greta did not share meat with any individual.

At 13:15, Chenge followed by an adult female, Roxy, climbed up the tree. Roxy moved toward Greta and took the body. She then began to consume the hands and internal organs of the gorilla infant. Between 13:16 and 14:00, Roxy allowed Chenge, Littlegrey, Onome, Orian, and Queliba to access small amounts of meat, no begging behaviour was observed between individuals. A final gorilla chest beat was heard at 13:46 in a North-East direction of the observers.

At 14:10, Roxy climbed down the tree carrying the body, and began travelling South-East. She continued to occasionally feed on the body throughout the remainder of the afternoon and though some individuals approached to peer at the infant gorilla, no further food sharing behaviours were observed. At 17:15, the remains of the infant gorilla were abandoned by Roxy. Most of the internal organs, both legs and the brain had been consumed (for further details see Supplementary Information).

## Discussion

Here, we report the first observations of two lethal coalitionary attacks of chimpanzees on another hominid species, gorillas. In both events, the chimpanzees considerably outnumbered the gorillas, however in the second event, the lethal attack started when the silverback had abandoned his group. In both events, the victims were gorilla infants, but the consumption of the victim was observed in one event only.

Recent studies^59^ were able to distinguish genetically distinct gorilla groups within the study area; when overlaid against the Rekambo chimpanzee community home range there was clear overlap with seven distinct gorilla groups (see Supplementary Fig. S1 in the Supplementary information). However, further data are needed to clarify whether our rare observations are due to lack of data or indeed mirror true frequencies of interspecies interactions in the study area.

In the following paragraphs, we will present and discuss several possible explanations that may account for the two lethal coalitionary encounters observed.

One explanation may be that the observed events represent cases of predation with the chimpanzees hunting and opportunistically targeting the smaller-bodied gorilla infants as prey. Although differences in behaviours accompanying hunting and hunting patrol patterns have been observed across sites, the behaviors observed at Loango were similar to the patterns reported for Taï^41,60^ and Ngogo^38^. For instance, the chimpanzees showed conspicuous behaviours prior to hunting such as being extremely attentive to any arboreal movements, scanning, changing directions several times without vocalizing, and performing specific call types—hunting calls^38,45^. Post-hunting behaviour is characterized by the prevalence of high-ranking males as the primary prey possessors and consumers, high levels of attention, arousal and excitement of party members, as well as begging and food sharing^13,40–41,61^. However, the behaviours observed during the two events were very different to those reported during hunting: The chimpanzees were noisy, emitted alarm barks and screams and performed displays long before the infants were killed. The excitement levels dropped immediately following the death of the infant gorillas. In addition, the observed feeding behaviours during the two events also differed from patterns expected during conventional hunting for the purpose of gaining nutritional benefits through the consumption of prey^27^. In the first encounter no feeding behaviour was observed, and in the second event the gorilla infant was almost entirely consumed by a single adult female. In contrast to species-typical hunts, in the second event the majority of individuals present, including adult males, showed almost no interest in the carcass, and only small amounts of meat were exchanged between low ranking individuals.

Another explanation may be that the two cases are the product of interspecific competition such as IGP and IK. So far, studies investigating interspecific competition in gorillas and chimpanzees have provided evidence for dietary niche differentiation and mutual avoidance to limit competition e.g.,^62–67^. All previous accounts of interspecies interactions as well as co-feeding events have been reported as peaceful despite a relatively high potential for feeding competition concerning key resources or during certain periods e.g.,^68–70^. Thus far, aggressive interference competition, including infanticide, has been observed between monkey species (e.g., Cercopithecus nicitans stampflii, Cercopithecus diana diana^71^; Ateles hybridus, Alouatta seniculus^72^) but not between chimpanzees and gorillas. Such interactions are however frequent in carnivore species and have been suggested as key determinants of their abundance and distribution^33,73^ (but see for an overview of other taxa^31^). As in the lethal interactions discussed here, carnivores tend to attack their closest dietary competitors^31^, most agonistic encounters occur in seasonal environments when food is scarce^27^, and killings decrease abruptly when dietary overlap is reduced^73^. Gorillas and chimpanzees show considerable dietary overlap and have a relatively high potential for dietary competition^45,74^. Across study communities, the degree of dietary overlap ranges between: 50% Kahuzi-Biega; Gorilla beringei graueri, Pan troglodytes schweinfurthii^75^ and 60–80% Loango, Lopé, and Ndoki; Gorilla g. gorilla, Pan t. troglodytes^66,74,76^. The two lethal encounters we observed occurred at times characterized by food scarcity and a period of high dietary overlap (for fruit resources)^45,74^—February and December 2019. In contrast, the two previously observed peaceful co-feeding events took place in April, a month characterized by relatively low dietary overlap between the two species^45,74^.

Furthermore, age, size and patterns of grouping seem to play a significant role in the outcome of IGP’s and IK’s (see e.g.,^27^). While relative body size of the opponents is the primary determinant of lethal interactions and results in favour of the larger species, in interactions involving adults, smaller species frequently kill the young of larger species^27,73^. There are cases where smaller species were able to kill or deter larger species such as wolves (*Canis lupus*) killing adult black bears (*Ursus americanus*)^77^ and hyenas (*Crocuta Crocuta*) killing lions (*Panthera leo*)^27^, however, these outcomes were only possible when individuals of the smaller species formed coalitions^27,77^. The grouping style of a species was found to strongly influence the outcome of IGP’s resulting largely in favour of species that form groups^78^. This is in line with our current observations, where the chimpanzees were at an advantage even against the larger gorilla species, given their ability to cooperate. Additionally, specific adaptations to prey-capture also influence the outcome of IGP’s, resulting in favour of species more adapted for vertebrate predation^73^ where the successful species, here, the chimpanzee, has adaptations to vertebrate predation^13,39,41^. Hence, as in IGP food webs (with specific emphasis of species classification) portrayed by Arim and Marquet^79^, the two reported killings may represent cases of IGP and IK between an intermediate omnivorous species (i.e. broad diets comprising both animal and plant foods^80^), the chimpanzee, and a herbivorous species (feeding mainly on plant foods^81^), the gorilla.

Lastly, both of the lethal encounters reported here also showed similarities to behaviours observed during chimpanzee intercommunity encounters. For instance, similar to territorial patrols, where chimpanzees move to the periphery and beyond their territorial boundaries to search for neighbours e.g.,^11,13,82,83^, the observed events took place in the peripheries of the territory before and during territorial patrols. In both events, infants were targeted and adult males were the main attackers and played the most active roles. Similarly, in lethal chimpanzee intercommunity encounters, infanticide is common and adult males are the main participants^11,51,83,84^ (but see for female roles^51^). It has been proposed that in chimpanzees, adult males may kill infants of other communities to reduce competition for food by inducing foreign females to avoid contested regions^84^. The observed interspecies killings of gorilla infants by chimpanzees could have similar motivations^85^. We also observed behaviours before and during the encounters characteristic to coalitionary intercommunity encounters such as aggression (e.g., charges, chases, threatening displays, contact aggression), high levels of arousal and the use of loud vocalizations^13–15,51^. The imbalance-of-power hypothesis postulates that the function of unprovoked intercommunity aggression (such as deep incursions into other chimpanzee communities’ territory and coalitionary attacks) is a drive for dominance over neighbours resulting in fitness benefits for the attackers through improved access to resources such as food, females, or safety^6,13^. Two conditions are proposed to be required to trigger coalitional killing of neighbours: (i) a state of intergroup hostility, and (ii) sufficient imbalances of power between interacting parties resulting in impunity from aggressors. Thus, it may be possible that at Loango, which is characterized by relatively high dietary food overlap in specific months^45,74^, gorillas are perceived as competitors, for both space and resource use, similar to members of other chimpanzee communities. Lastly, we cannot rule out that the presence of human observers, in both events, may have had an effect on the unhabituated silverback’s departure and may have tilted the imbalance of power in favour of the habituated chimpanzees.

In sum, the observed events show similarities to patterns reported in IGP’s, IK’s and intraspecies agonistic encounters. Ultimately, additional observations in combination with isochronous assessments of fruit availability and dietary overlap are needed to differentiate whether coalitionary attacks are indeed the output of interspecific predation spurred by opportunistic hunting, interspecies competition for food resources or whether these interactions are merely a non-adaptive by-product of the “xenophobic nature” of chimpanzees. Finally, analyses of long-term phenological data could aid in investigating if potential high levels of feeding competition may be a more recent phenomenon caused by a collapse in fruit availability as observed in other tropical forests in Gabon^86^.

## Conclusion

Our observations provide the first evidence that the presence of chimpanzees can have a lethal impact on gorillas. Additionally, they may instigate future studies aiming to test whether lethal coalitionary interactions of chimpanzees against gorillas are the output of opportunistic hunting or interspecies competition. Future studies could try to simultaneously monitor movement patterns and monthly dietary overlap of the two species in relation to actual encounter rates and outcomes. Future investigations of interspecific interactions may also encourage more cross-fertilization between behavioural ecologists and palaeontologists^87,88^. Gorillas and chimpanzees have adapted to sympatry throughout their own evolutionary histories. However, up until now, their extensive population decrease and the threats of extinction have predominantly been driven by escalating anthropogenic pressures rather than by the presence of another non-human great ape species^89^. Ultimately, these living models, combined with continuous new insights from the fossil record, can aid in expanding our understanding of the ecological constraints and mechanisms governing the co-existence of not only these two ape species but other, now extinct, sympatric hominin taxa.

## Methods

The two lethal encounters were observed whilst conducting behavioural observations on chimpanzees of the Rekambo community in the Loango National Park, Gabon (2° 04′ S and 9° 33′ E). The habituation of this community began in 2005 with the majority of individuals being habituated to human presence by 2017. The community consisted of about 45 individuals including infants, juveniles and sub-adults at the end of the data collection period of the present study. The Loango National Park comprises a mosaic of different habitat types including coastal forests and savannah in the West, multiple lowland swamps fed by a lagoon in the East, and heterogeneous tropical rainforest throughout (for further details see^74^). This ecosystem is considerably different from other locations where sympatric ape species have been studied so far^63,74^. Information concerning territorial overlap between the two species at the study site can be found in Supplementary Fig. S1 in the Supplementary information.

Focal animal sampling was used during daily follows of all mature males (N = 11). Recordings were made using high-definition video cameras (SONY AX53) with external microphones (Sennheiser ME400). We used all occurence sampling during the interspecies encounters^90^. Data collection was operationalized with CyberTracker software (CyberTracker version 3.507; https://www.cybertracker.org/)^91^ on water-resistant smart phones (Cyrus CS45). In addition to the behavioural data collection, daily tracklog data were recorded using a Global Positioning System (GPS; Garmin Rino700) which records a given location automatically every 1–60 s depending on signal coverage. Additionally, GPS coordinates were recorded whenever an interspecies interaction was detected. We calculated territory size by collating all track logs (> 1 h) using the Kernel Density Estimation (KDE) method using the package adehabitatHR^57^ in R (version 3.6.3, R Core team, https://www.r-project.org/)^56^ Fixed kernel density estimation was generated using the reference method (*h*_ref_) to provide contours of utilization of 75% and 50%. We created a map (see Fig. 1) depicting the 75% and 50% KDEs, together with data on both non-lethal encounter points from 2014 to 2019 and lethal encounter locations using R. Furthermore, we depicted the territories of gorilla groups at the study site and potential overlap with the territory of the Rekambo community in a map (see Supplementary Fig. S1 in the Supplementary Information) using the MCP method for the Rekambo chimpanzee territory and published data from Hagemann et al.^59^ concerning gorilla territories at and overlapping with the study area.

### Ethics statement

The present study was purely observational and non-invasive. All applicable national, and/or institutional guidelines for the care and use of animals were followed. In accordance with the German Animal Welfare Act of 25th May 1998, Section V, Article 7, the study was classified as non-animal experiment and did not require any approval from a relevant body. All observers followed a strict hygiene protocol, including a five-day quarantine, and wore face masks when encountering chimpanzees. Observations were made at a minimum distance of eight meters, in an effort to avoid disease transmission from humans to chimpanzees^92,93^ and to not disturb the natural behaviour of the individuals observed. Our research adhered to the legal requirements of the state of Gabon and followed the recommendations of the ‘Animals (Scientific Procedures) Act 1986’, as published by the government of the United Kingdom, and the principles of “Ethical Treatment of Non-Human Primates”, as stated by the American Society of Primatologists. The Agence Nationale des Parcs Nationaux, and the Centre National de la Recherche Scientifique et Technique of Gabon (CENAREST), Libreville, Gabon granted permission and the relevant permits to conduct research in the Loango National Park.

## Supplementary Information

Supplementary Video 1.

Supplementary Video 2.

Supplementary Information.

Supplementary Legends.
