# Supplementary material for: Lethal coalitionary attacks of chimpanzees (Pan troglodytes troglodytes) on gorillas (Gorilla gorilla gorilla) in the wild
Source: Sci Rep. 2021 Jul 19;11:14673. doi: 10.1038/s41598-021-93829-x (PMC8290027; doi:10.1038/s41598-021-93829-x)
Supplement: Supplementary file 3 — Supplementary Information. [file 41598_2021_93829_MOESM3_ESM.docx]

**Lethal coalitionary attacks of chimpanzees (*Pan troglodytes troglodytes)* on gorillas *(Gorilla gorilla gorilla*) in the wild**

**Lara Michelle Southern^1, 2^, Tobias Deschner^2, 3^, Simone Pika^1,3,*^**

^1^University of Osnabrück, Institute of Cognitive Science, Comparative BioCognition, Artilleriestrasse 34, 49076 Osnabrück, Germany

^2^Max Planck Institute for Evolutionary Anthropology, Interim Group Primatology, Deutscher Platz 6, 04103, Leipzig, Germany

^3^shared last authorship

*Please address all correspondence to Simone Pika: [spika@uos.de](mailto:spika@uos.de)

**Supplementary material**

***Detailed Reports***

***Report 1***

**Date:** 6^th^ February 2019

**Observers:** Alessandra Mascaro, Freddy Makaya, Jean Edouard Nzengue, Lara Michelle Southern and Marcoso Etembe

**Location:** 2°07'33.9"S 9°30'17.4"E

**Duration:** 17:01 – 17:53 (52 minutes total: 15 minutes visual contact**,** 37 minutes auditory contact)

**Observed gorilla group composition**: One silverback, three adult females, one infant

**Chimpanzee party composition:** 27 individuals

| **Individual** | **Sex** | **Age class** | **Role during encounter 1** | **Role during encounter 2** |
| --- | --- | --- | --- | --- |
| Chenge | M | adult | active (APC) | active (APC) |
| Chinois | M | adult | active (NPC) | active (NPC) |
| Freddy | M | adult | active (APC) | active (APC) |
| Littlegrey | M | adult | active (APC) | active (APC) |
| Louis | M | adult | active (APC) | active (NPC) |
| Pandi | M | adult | active (APC) | active (APC) |
| Thea | M | adult | active (APC) | active (APC) |
| Carol | F | adult | not active | not active |
| Emmie | F | adult | N/A | not involved |
| Ida | F | adult | not involved | N/A |
| Joy | F | adult | not involved | not involved |
| Mimi | F | adult | not involved | active (APC) |
| Onome | F | adult | not involved | not involved |
| Roxy | F | adult | active (APC) | active (APC) |
| Spock | F | adult | N/A | not involved |
| Suzee | F | adult | not involved | N/A |
| Arnold | M | adolescent | not active | active (APC) |
| Gump | M | adolescent | active (APC) | active (APC) |
| Ngonde | M | adolescent | active (APC) | active (APC) |
| Orian | M | adolescent | not active | active (APC) |
| Clessia | F | adolescent | active (PC) | N/A |
| Gia | F | adolescent | active (APC) | active (PC) |
| Greta | F | adolescent | N/A | active (PC) |
| Harouenne | F | adolescent | active (PC) | N/A |
| Queliba | F | adolescent | N/A | active (PC) |
| Cesar | M | juvenile | active (PC) | active (PC) |
| Ernest | M | juvenile | N/A | not involved |
| Moana | M | juvenile | not involved | active (PC) |
| Sia | M | juvenile | active (PC) | N/A |
| Ismael | M | infant | not involved | N/A |
| Jambo | M | infant | N/A | not involved |
| Caya | F | infant | N/A | not active |
| Madiba | F | infant | not involved | not active |
| Sassandra | F | infant | not involved | N/A |

**Table S1. Chimpanzees observed during the two gorilla encounters.** Members of the Rekambo chimpanzee community present during encounter 1 (N=27) and encounter 2 (N=27) as a function of name, sex (M= male; F= female), age class and role. The role describes the observed activity of individuals during the encounters: N/A: not present; *not involved* – seen at the start of the encounter and following but no direct (either vocal or contact) interspecies interaction, presence is inferred; *not active* – observed during the encounter but no active role in the attack; *active-no physical contact (NPC)* – active role in the attack (e.g. charging, chasing) but no direct contact with a gorilla; *active-aggressive physical contact (APC)* – active role in the attack with direct aggressive behaviors towards a gorilla individual (e.g. hitting, biting, dragging); *active-physical contact (PC)* – present throughout attack but direct contact only through handling already deceased gorilla (e.g. touching, sniffing, handling).

**Overview:** On the 6^th^ of February 2019, 27 members of the Rekambo community (see table S1), went on a deep intrusion patrol. They patrolled in a zone to the far east of their territory where human observers had not yet followed the group (since the habituation of the community in 2017). The zone consists predominantly of inundated swamp forest, primary and secondary forest. During the patrol, all individuals remained completely silent with the exception of the use of soft food grunts after arriving at two separate feeding sites of *Strombosia glaucesens*. The group re-entered the outskirts of their territory at 16:45 (see figure 1) where they began to pant hoot and drum. The encounter with the gorilla group took place near the border of what we consider the Rekambo territory at 17:01.

**6^th^ February 2019:** At 16:58, Team 1 (FM, AM, and JN) had been following a party of nine chimpanzees including two adult males (Chinois and Louis), three sub-adult males (Arnold, Gump and Ngonde), one adult female (Suzee with her offspring), one juvenile male (Sia) and an infant female (Sassandra). Two males (Louis and Ngonde) had been feeding in a *Strombosia* *glaucesens* tree for approximately three minutes (16:58 - 17:01.) while the other individuals rested on the ground. Then the observers heard two separate sub parties of chimpanzees at approximately 200m and 300m to the north-west and to the south-west respectively. At 17:01, a loud high-pitched scream of a chimpanzee was heard at a distance of about 100m to the west followed by a series of chimpanzee barks. Twenty seconds later there was a series of loud screams and barks from the same direction. Hearing these screams, Louis and Ngonde quickly climbed down the tree, while the other individuals had already run into the direction of the barks. Louis started a display, tearing at the vegetation around him whilst pant hooting, Ngonde followed him without displaying or pant hooting. The two males did a small circle around a fallen trunk and then charged into the ‘thicket’, at the source of all the commotion. At the same time, team 1 heard the first gorilla bark originating from the commotion and subsequently observed one silverback, and two adult female gorillas in the thicket.

Team 2 (LMS and ME) were situated on the opposite side of the ‘thicket’ since they had been following another adult male, Pandi. Prior to 17:01, Pandi had been travelling with Carol, Clessia and Cesar (see table S1) and other chimpanzees of the Rekambo community in the surrounding 50m. Upon hearing the initial scream at 17:01, Pandi immediately moved into that direction and disappeared into the thicket. A few seconds later, loud barks and screams started. Upon hearing the gorilla barks and chest beats, team 2 decided to move around the thicket to find a safe observation point. At 17:03, the two observation teams reunited and moved next to a big fallen log where they were able to listen to the commotion from a safer distance (approximately 15m from the thicket). The two teams positioned themselves at different angles to the thicket to maximize observation possibilities. At this time, the teams were able to observe one silverback and at least three adult female gorillas. However, it was unclear whether the females had infants with them. The loud commotion lasted approximately ten minutes (17:02 - 17:12) during which multiple male chimpanzees (Freddy, Littlegrey, Louis, Pandi, Thea, Gump and Ngonde) and the silverback charged in and out of the thicket. Due to the nature of the dense habitat, visual observations were limited for both observation teams. At 17:13, team 1 observed the silverback charging an adolescent female chimpanzee, Gia, and knocking her into the air. At 17:15, a group of approximately eight adult and adolescent males and at least one adult female chimpanzee surrounded the silverback in the lianas within the thicket. They repeatedly jumped down on and hit the silverback whilst screaming and barking. No female gorillas were observed during the contact aggressions between the chimpanzees and the silverback. At 17:16, the silverback retreated to a distance of approximately 30m with all other members of his group. After the retreat, no other adult gorilla was seen for the remaining duration of the encounter. At 17:17, the observers approached the chimpanzee party to a distance of 10m. At 17:22, one adult male, Littlegrey, was observed holding a gorilla infant in front of him. The infant omitted distress vocalizations but did not move. Thea, Gump, Ngonde and Clessia were sitting around him, and peered at his hands and the gorilla infant. When Littlegrey changed his position, team 1 observed that he was holding down an infant gorilla that had not been observed before. At 17:23, Gump took the infant gorilla from Littlegrey and pulled it behind him. Littlegrey, Ngonde and Clessia followed him. When Gump began to pull the infant, all other chimpanzees present joined in a pant hoot chorus (identified individuals that joined included Chinois, Freddy, Louis, Pandi, Thea, Gump, Ngonde and Harouenne). Gump then moved into a closed part of the thicket resulting in the human observers losing visual contact. At 17:24, they regained sight of the gorilla infant, which was now held and sniffed by Littlegrey. Ngonde, Clessia and Cesar observed Littlegrey and the infant from a distance of approximately one meter. He held the infant and smelt it two times. Then Clessia approached the infant, put her face close to the gorilla’s anus and sniffed. At 17:26, Littlegrey sniffed at the infant, held it down on the ground with his left hand and hit it three times with his right hand. He then pressed his body weight on the infant. At this point the gorilla infant was still alive since short squeaks and whimpering sounds could be heard. At 17:26 and in response to the soft squeaks and whimpers from the infant, a whimper and a cry from a non-visible gorilla in a distance of approximately 20-30m was heard. At 17:27, Ngonde approached Littlegrey, grabbed the gorilla infant and left with it. Littlegrey did not respond, but Clessia and Cesar followed Ngonde. The observers regained sight of the gorilla infant being carried by Clessia at 17:28 Harouenne and Cesar were following her. At 17:28 and directly following a distress vocalization of the infant gorilla, another whimpering vocalization of a gorilla was heard (in the same location as before in a distance of approximately 20-30 meters from the chimpanzee party). At 17:28, Ngonde took the gorilla infant from Clessia and pulled it by its right foot on the ground for approximately three meters. The infant omitted distress vocalizations but remained motionless. At 17:30, Clessia, took the gorilla infant from Ngonde, sniffed the body, and held it with both hands while lying on her back. She held the gorilla on her belly, pulled its feet, and played with it. Harouenne, Cesar and Sia were within one meter of Clessia and the infant and watched her. At 17:32 and in response to the continuous whimpering of the infant gorilla, another whimper and cry was heard from an out-of-sight gorilla (again at a distance of approximately 20-30m). At 05:36, the gorilla infant stopped vocalizing, and no further vital signs were observed. Between 17:36-17:53, Clessia continued to hold and play with the now lifeless body of the gorilla periodically. At 17:53, a chest beat was heard from a distance of approximately 40m, but subsequently no further sound, vocalization or indication of gorilla presence was noticed. Between 17:53 and 17:59, Clessia remained in body contact with the dead infant while occasionally playing with one of its legs or arms. During this time no other individual approached her or peered at the dead body. The other chimpanzee individuals were resting on the ground in and around the thicket. At 17:59, the first chimpanzee individuals started to leave the thicket (heading North) in the opposite direction that the gorillas had left (South). Littlegrey, Louis, Thea, Gump, Ngonde, Joy, Suzee, Harouenne and Sia were the first individuals seen climbing up a *Strombosia glaucesens* tree approximately 25 meters north of the encounter site. Due to the advanced time, the human observers were obliged to leave the party. At 18:06, Clessia was the last individual seen with the body of the gorilla infant, sitting holding it on the ground.

**7^th^ and 8^th^ February 2019.** The next morning (02/07/2019) at 07:00, a party of chimpanzees (Louis, Thea, Arnold, Gump, Ngonde, Orian, Joy, Onome, and Harouenne) were located at the same *Strombosia glaucacens* tree where the two observer teams had left the group the evening before. At 07:09, all individuals climbed down the tree and began moving North. There was a fission of two chimpanzee parties at 09:10 where Chenge, Freddy, Littlegrey, Pandi, Roxy and Gia joined the original group. Pandi was observed with a cut (of approximately 3 cm) on his right side and a large scratch on his right cheek. Freddy had a deep cut (of approximately 5 cm) on his back with bloody matted fur around the opening. Gia was limping and could not put any pressure on her right leg. She was also very lethargic and stayed behind the group the whole day struggling to keep up with the travelling speed. Team 2 (JEN and LMS) went back to the location of the encounter and searched for the body of the infant gorilla. Unfortunately, the body was not found. Furthermore, Clessia (the last individual observed with the body of the gorilla) was only observed three days later (10/02/2019).

On February 8^th^, two observer teams did another full sweep of the area with a margin of 200m around the thicket where the gorilla encounter took place. However, no body, nor body remains of the dead infant gorilla were located.

***Report 2***

**Date:** 11^th^ December 2019

**Observers:** Alessandra Mascaro, Freddy Makaya,, Jean Edouard Nzengue, Justin Kombe, Lara Michelle Southern and Viola Haring

**Location:** 2°03'51.0"S 9°28'50.6"E

**Duration:** 12:27-13:46 (79 minutes total: 26 minutes visual contact**,** 53 minutes auditory contact)

**Observed gorilla group composition:** One silverback, two adult females, one juvenile, two infants

**Chimpanzee party composition:** 27 individuals

**Observations of the first (JK and LMS) and second team (AM and FM).** On December 11^th^ 2019, four observers, FM, AM, JK, and LMS had been following a large group of 27 chimpanzees since 07:20 (see table S1). The group had progressively been moving towards the edge of their territory in the North (see figure 1 and S1). The first team (JK and LMS) were following an adult male, Freddy, and the second team (FM and AM) were following an adult female, Joy. At 12:26, Freddy suddenly stopped, became pilo-erect, and produced alarm barks whilst looking into the distance towards the edge of a swamp. The chimpanzees around him stopped travelling and looked in the same direction while also emitting alarm barks in chorus. Searching for the cause of these barks, JK and LMS saw movements in a large tree in a distance of approximately 40m. At 12:28, they identified an adult female gorilla in the canopy at a height of approximately 30m. The chimpanzees continued to alarm bark, while simultaneously moving towards the tree. One adult female gorilla was observed to climb down the tree, fleeing west along the edge of a swamp and in the opposite direction of the approaching chimpanzees. The chimpanzees observed her, and continued barking, but did not follow her. Instead, they moved closer to the base of the tree. At 12:28, JK and LMS heard gorilla barks and chest beats, and observed six additional gorillas in the tree: a silverback, two adult females with dependent infants, and one juvenile gorilla. At 12:30, the majority of the chimpanzees started to climb up into the surrounding trees, while approximately four adult male chimpanzees remained on the ground. At 12:33, the silverback moved onto a large branch of the tree and discovered the four human observers (JK, FM, AM, and LMS) on the ground in a distance of approximately 30m. He started to bark and beat against the branch he was sitting on. In response, the observers increased their distance to the feeding tree from 30m to 60m. One adult male chimpanzee, Chenge, climbed up the tree with the gorillas and stopped within five meters of the silverback and one adult female gorilla with an infant (AF1 and I1). The second female with an infant sat five meters above the silverback. All visible gorillas started to emit alarm barks, and the silverback and the two adult females with their infants moved higher up into the canopy. Chenge followed them along with other chimpanzees a distance of five to ten meters while barking continuously and looking up into the canopy. The silverback barked again and produced chest beats while the chimpanzees continued to scream and bark. At 12:36, the silverback rapidly climbed down the tree and left the location running in a South-West direction. The chimpanzees continued barking but no individual followed him. At 12:37, JK and LMS saw one of the two adult gorilla females (AF2) with her infant (EI2) on her belly starting to climb down the tree with a group of chimpanzees (composed of the individuals that were lower in the same tree and those in the surrounding trees) surrounding her while barking, screaming, charging and branch shaking. Once she reached the ground, the group of chimpanzees including mainly adult chimpanzee males (Chenge, Freddy, Littlegrey, Louis, Pandi, Thea), but also two adult females (Roxy and Mimi), sub adults (Arnold, Gump, Ngonde, Orian) and a juvenile (Moana) surrounded her. Several non-contact aggressions occurred, mostly involving purely visual charges and branch shaking. Pandi and Thea used branches from the surrounding bushes and displayed at the female. She held her infant on her belly and barked, while the infant was also barking and screaming. Until 12:38, the chimpanzees repeatedly barked and displayed at the adult gorilla female. However, there was still relatively little physical contact. For instance, Thea, who was repeatedly observed directly in front of the female, tried to grab the infant but did not succeed. In addition, Gump tried to pull the infant from the belly of the female but she managed to pull it back and returned the infant to her belly. At 12:40, the female managed to leave the cluster of chimpanzees, with several individuals engaging in within-species aggressions (e.g., Pandi was seen to charge Littlegrey). In addition, the chimpanzees directed their attention to an area approximately 20m away across a swamp where gorilla screams and barks could be heard. At 12:40, the gorilla female (AF2 with EI2) was able to escape with her infant in a South-East direction. No chimpanzees were observed following them. Between 12:40 and 12:46, four of the human observers (JK, FM, AM, and LMS) lost visual contact with the group and could not observe details of the second attack. However, they were able to record the vocalizations and sounds accompanying the second attack. The third team of observers (VH and JEN) witnessed the second contact aggression but visibility was very low (see details below).

**Observations of the third team (VH and JEN).** The third team (VH and JEN) were following an adult male, Chinois. He was resting at a distance of 400m from the larger party followed by the first observer team. After hearing the loud vocalizations at 12:30, he moved rapidly in that direction whilst displaying on the way. At 12:33, he arrived at the scene of the encounter, and VH and JEN observed at least three gorillas (unknown sex but subadult/adult individuals) on the ground fleeing towards the East. At this point, the two observers lost visual contact with Chinois but were able to see the same aggression encounter between chimpanzees and the gorilla female AF2 and EI2 (detailed above) from a different perspective.

Between 12:41-12:48, five adult chimpanzee males (Chenge, Littlegrey, Louis, Pandi, Thea), and four adolescent individuals (Gump, Greta, Ngonde, Orian) were sitting in several trees in the middle of a swamp. One adult female gorilla (AF1) with a smaller infant (I1) was seen in a tree surrounded by several chimpanzees in the trees around her. No other gorilla was visible at this point; however, chest beats and barks were heard from the surrounding canopy in the forest around the swamp. Littlegrey, Louis, and Pandi displayed repeatedly shaking the branches in the trees around them, and all chimpanzees continuously emitted alarm barks. The gorilla female remained hidden in the canopy but uttered screams and barks as soon as any chimpanzee undercut a distance of, approximately five meters. At 12:48, four chimpanzee males (Ngonde, Orian, Pandi and Thea) started to chase the female first up a tree, and then down the tree while barking and screaming. During approaches, the gorilla female (AF1) waved her arms and screamed at the chimpanzee males, whilst holding her infant (I1) to her belly, and simultaneously trying to move out of reaching distance of the chimpanzees. At 12:49, the gorilla female managed to flee with her infant on her belly down the tree into a tangle of lianas on the swamp bed. The two observers (VH and JEN) could not see any direct interactions within the lianas but heard barks and screams. At 12:50, the gorilla female (AF1) was seen, without her infant (I1), climbing up a tree and being followed by several chimpanzees (Freddy, Gump, Louis, Mimi, Moana, and Thea). At 12:50, she was observed climbing into a higher tree and moving away in the canopy towards the East. All chimpanzees climbed down the surrounding trees, and continued to move towards the other side of the swamp (North). At 12:51, a juvenile chimpanzee, Cesar, was seen holding the body of the dead infant gorilla (I1), which had a large open cut in the stomach with the intestines visible from the exterior. Cesar took the body and climbed up a tree, while holding the body with his left hand and swinging it beside him. At 12:51, he climbed down the tree still holding the body of the gorilla infant. Gump approached him and, grabbed the body, and ran away with it. At 12:53, chest beats of a gorilla were heard at a distance of approximately 75m North-East of the observers. At this time, all other chimpanzees of the party were dispersed around the encounter site, and rested either on the ground or up in the trees. No individual reacted to the gorilla vocalizations. The observers were able to locate the body of the gorilla infant again at 12:57, when Gump, followed by Greta, climbed up a tree still holding it. At 13:00, one adult female (Onome) and two adolescent females (Greta and Queliba) were observed in a tree. Greta was now holding the body. Although all three individuals were within reach of the body, only Greta was observed eating small pieces of meat from the extremities. She did not share meat with the other females. At 13:15, Chenge followed by an adult female, Roxy, climbed up the tree. Subsequently, Roxy moved toward Greta and took the body with no visual or acoustic protest from her or other individuals. She then began to feed, first eating the hands and then the internal organs. At 13:16, Onome took a small piece of meat from the gash in the stomach with no protest from Roxy. At 13:18., Littlegrey arrived and waited on a lower branch of the tree. At 013:20, he approached Roxy, licked some blood and took a small piece of meat from the infant’s foot. At 13:22, Orian arrived and took a piece of meat from Onome’s mouth, that Onome had previously taken from Roxy. Between 13:20 and 14:00, Roxy shared small amounts of meat actively with Chenge and passively with Littlegrey, Onome, Orian, and Queliba. A final gorilla chest beat was heard at 13:46 in North-East direction of the observers. At 14:10, Roxy climbed down the tree carrying the body and travelled towards the South-East. The other individuals followed her, peered at the body but did not try to take the body away from Roxy. She continued to occasionally feed on the body throughout the remainder of the afternoon but no further food sharing behaviors were observed. At 17:15, Roxy left the body of the infant on the ground after having fed on fruits of *Irvingia gabonensis*. Though most of the internal organs, both legs and the brain had been consumed, VH collected the body and brought it back to camp to perform an autopsy.


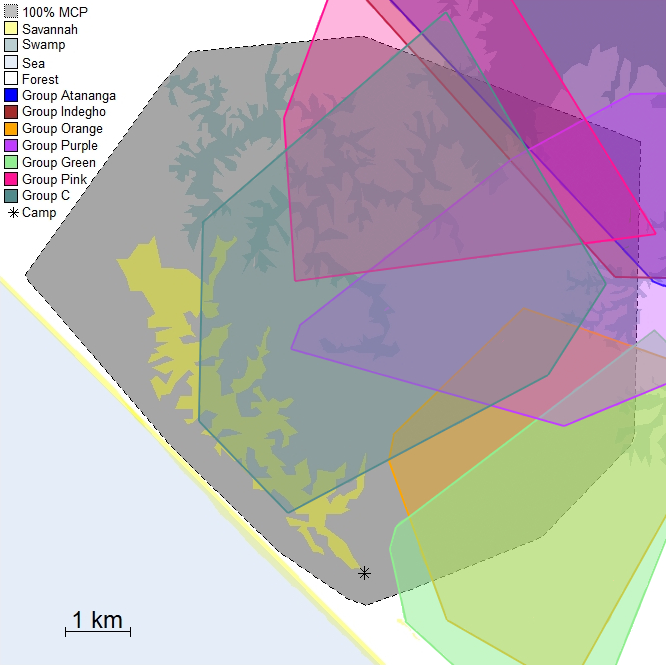


**Figure S1. Overlap between the Rekambo community 100% MCP and MCPs of seven other gorilla groups within the study area.** The Rekambo MC P is indicated by the dashed line. The seven other gorilla MCPs are colour coded in the legend. Data for the gorilla MCPs was taken from a published figure of Hagemann and colleagues [1].

1. Hagemann, L., Arandjelovic, M., Robbins, M.M., Deschner, T., Lewis, M., Froese, G., Boesch, C., and Vigilant, L. (2019). Long-term inference of population size and habitat use in a socially dynamic population of wild western lowland gorillas. Conservation Genetics *20*, 1303-1314.
