# Supplementary material for: Lethal coalitionary attacks of chimpanzees (Pan troglodytes troglodytes) on gorillas (Gorilla gorilla gorilla) in the wild
Source: Sci Rep. 2021 Jul 19;11:14673. doi: 10.1038/s41598-021-93829-x (PMC8290027; doi:10.1038/s41598-021-93829-x)
Supplement: Supplementary file 4 — Supplementary Legends. [file 41598_2021_93829_MOESM4_ESM.docx]

**Supplementary Video Legends**

Supplementary Video 1: Chimpanzees attacking gorilla mother with her infant; scene from encounter 1 (06/02/2019)

Supplementary Video 2: Chimpanzees attacking gorilla mother with her infant; scene from encounter 2 (11/12/2019)
